# Mefatinib as first-line treatment of patients with advanced *EGFR*-mutant non-small-cell lung cancer: a phase Ib/II efficacy and biomarker study

**DOI:** 10.1038/s41392-021-00773-3

**Published:** 2021-11-01

**Authors:** Pingli Wang, Yuping Li, Dongqing Lv, Lingge Yang, Liren Ding, Jianya Zhou, Wei Hong, Youfei Chen, Dongqing Zhang, Susu He, Jianying Zhou, Kai Wang

**Affiliations:** 1 Department of Respiratory and Critical Care Medicine, The Second Affiliated Hospital of Zhejiang University School of Medicine, Hangzhou, China; 2 Department of Respiratory and Critical Care Medicine, The First Affiliated Hospital of Wenzhou Medical University, Wenzhou, China; 3 Department of Respiratory Medicine, Taizhou Hospital of Zhejiang Province Affiliated to Wenzhou Medical University, Linhai, China; 4 Department of Respiratory and Critical Care Medicine, The Fourth Affiliated Hospital of Zhejiang University School of Medicine, Yiwu, China; 5 Department of Respiratory Diseases, Thoracic Disease Diagnosis and Treatment Center, The First Affiliated Hospital, College of Medicine, Zhejiang University, Hangzhou, China; 6 Department of Oncology and Chemotherapy, Zhejiang Cancer Hospital, Hangzhou, China

**Keywords:** Lung cancer, Cancer genetics

## Abstract

EGFR inhibitors have revolutionized the treatment of advanced non-small-cell lung cancer (NSCLC). Mefatinib is a novel, bioavailable, second-generation, irreversible pan-*EGFR* inhibitor. This phase Ib/II open-label, single-arm, multi-center study investigated the efficacy, safety, biomarker, and resistance mechanisms of mefatinib in the first-line treatment of patients with advanced *EGFR*-mutant NSCLC. This study included 106 patients with *EGFR*-mutant stage IIIB-IV NSCLC who received first-line mefatinib at a daily dose of either 60 mg (*n* = 51) or 80 mg (*n* = 55). The primary endpoint was progression-free survival (PFS). Secondary endpoints were overall response rate (ORR), disease control rate (DCR), overall survival (OS), and safety. The cohort achieved an ORR of 84.9% and DCR of 97.2%. The median PFS was 15.4 months and the median OS was 31.6 months. Brain metastasis was detected in 29% of patients (*n* = 31) at diagnosis and demonstrated an ORR of 87.1%, PFS of 12.8 months, and OS of 25.2 months. Adverse events primarily involved skin and gastrointestinal toxicities, which were well-tolerated and manageable. Analyses of mutation profiles were performed using targeted sequencing of plasma samples at baseline, first follow-up 6 weeks from starting mefatinib therapy (F1), and at progression. Patients with concurrent *TP53* mutations had comparable PFS as wild-type *TP53* (14.0 vs 15.4 months; *p* = 0.315). Furthermore, circulating tumor DNA clearance was associated with longer PFS (*p* = 0.040) and OS (*p* = 0.002). *EGFR* T790M was the predominant molecular mechanism of mefatinib resistance (42.1%, 16/38). First-line mefatinib provides durable PFS and an acceptable toxicity profile in patients with advanced *EGFR*-mutant NSCLC.

## Introduction

Mutations in epidermal growth factor receptor (EGFR) has transformed the standard of care and improved the prognosis of a subset of patients whose lung cancers are driven primarily by EGFR.^1–8^ Numerous clinical trials have established the superiority of first-, second-, and third-generation EGFR-TKI in managing advanced *EGFR*-mutant non-small-cell lung cancer (NSCLC) compared with conventional chemotherapy regimens.^9–13^ In 2016, gefitinib was the first EGFR-TKI approved as first-line therapy of metastatic NSCLC harboring *EGFR* exon 19 deletion (19del) and exon 21 L858R.^9,13^ Secondary *EGFR* mutations, particularly T790M, are the most common mechanism of resistance acquired during EGFR-TKI treatment;^6,14,15^ however, some EGFR-TKI-resistant tumors that do not develop *EGFR* T790M mutations still retain their dependence on the EGFR pathway.^16,17^ Irreversible second-generation EGFR-TKIs, including afatinib and dacomitinib, were developed to overcome this resistance from reversible first-generation EGFR-TKIs gefitinib and erlotinib.^16–18^ Afatinib potently inhibits various ErbB receptor tyrosine kinase family members, including HER2 (ErbB2), ErbB3, and ErbB4, and was approved in the first-line treatment of patients with *EGFR*-mutant NSCLCs since 2013.^11,12,17,19,20^ As compared to gefitinib, afatinib as front-line therapy demonstrated a higher objective response rate (ORR, 72.5% vs 56%) and longer median time to treatment failure (13.7 vs 11.5 months) but no difference in overall survival (OS).^21,22^ On the other hand, dacomitinib as front-line therapy demonstrated a significantly improved progression-free survival (PFS, 14.7 vs. 9.2 months) and OS (OS, 34.1 vs. 27.0 months) compared with gefitinib.^18,23^ However, the presence of concurrent mutations, including *TP53* mutations and *EGFR* amplification, could severely limit the efficacy of currently available EGFR-TKI therapy, including afatinib, and lead to poorer clinical outcomes of patients harboring these concurrent mutations treated with EGFR-TKI.^24–28^

Mefatinib (MET306) is a novel, second-generation EGFR-TKI, which was designed to irreversibly bind to mutated tyrosine kinase domain of EGFR and HER2. Unpublished preclinical studies using xenograft mouse models have demonstrated the strong inhibitory activity of single-agent mefatinib on lung cancer harboring EGFR- or HER2-overexpression, or *EGFR* mutations.

In this article, we report the combined results of the phases Ib and II open-label, single-arm, multi-center studies on the clinical efficacy and safety of mefatinib in the first-line treatment of patients with advanced *EGFR*-mutant NSCLC. We also explored genetic biomarkers associated with the efficacy of mefatinib therapy and the molecular mechanisms mediating acquired resistance to mefatinib therapy.

## Results

### Patient characteristics

A total of 106 patients with advanced *EGFR*-mutant NSCLC were enrolled. The cohort comprised 43% males and 57% females with a median age of 64 years (range: 27–78 years). A majority of the cohort was never smokers (63.2%, 67/106) and 34.0% (36/106) were current and former smokers. All the patients were diagnosed with stage IIIB-IV lung adenocarcinoma and were EGFR-TKI-naive before receiving mefatinib therapy. The cohort also included 29.2% (31/106) patients who had brain metastasis at presentation. Before study inclusion, the *EGFR* mutation status of the patients was confirmed by ARMS-PCR of tissue biopsy samples, with 56.6% (60/106) of the patients harboring *EGFR* 19del, 40.6% (43/106) of the patients having *EGFR* L858R, and 3 (2.8%) patients having both mutations. Table 1 summarizes the baseline clinical characteristics of the patients.

**Table 1:** Baseline clinical characteristics of the enrolled patients

Clinical characteristics	All patients (*n* = 106)	60 mg (*n* = 51)	80 mg (*n* = 55)
Age (years) (median [range])	64 [27–78]	64 [27–78]	63 [37–77]
Sex			
Male	46 (43.4%)	22 (43.1%)	24 (43.6%)
Female	60 (56.6%)	29 (56.9%)	31 (56.4%)
ECOG PS			
0	48 (45.3%)	20 (39.2%)	28 (50.9%)
1	58 (54.7%)	31 (60.8%)	27 (49.1%)
Smoking history			
Current	10 (9.4%)	8 (15.7%)	2 (3.6%)
Former	26 (24.5%)	10 (19.6%)	16 (29.1%)
Never	67 (63.2%)	32 (62.7%)	35 (63.6%)
Unknown	3 (2.8%)	1 (2.0%)	2 (3.6%)
Disease stage			
IIIB	7 (6.6%)	1 (2.0%)	6 (10.9%)
IIIC	1 (0.9%)	1 (2.0%)	0 (0%)
IV	98 (92.5%)	49 (96.1%)	49 (89.1%)
Baseline brain metastasis			
Present	31 (29.2%)	16 (31.4%)	15 (27.3%)
Absent	74 (69.8%)	34 (66.7%)	40 (72.7%)
Unknown	1 (0.9%)	1 (2.0%)	0 (0%)
*EGFR* mutation status			
*EGFR* exon 21 L858R	60 (56.6%)	26 (51.0%)	34 (61.8%)
*EGFR* exon 19 deletion	43 (40.6%)	23 (45.1%)	20 (36.4%)
Both	3 (2.8%)	2 (3.9%)	1 (1.8%)

*ECOG PS* Eastern Cooperative Oncology Group performance score

### Efficacy

**Fig. 1:**
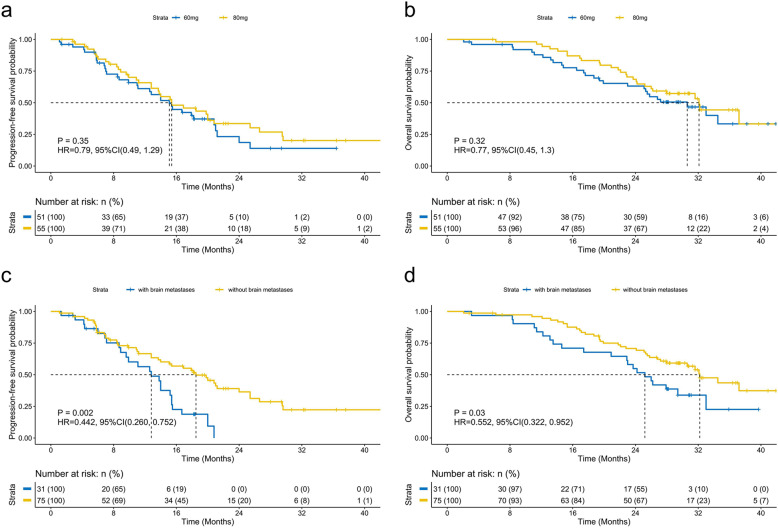
Survival outcomes with mefatinib were similar for patients who received mefatinib at 60 and 80 mg but were significantly different based on brain metastatic status at presentation. Kaplan–Meier curves comparing the progression-free survival (**a**, **c**) and overall survival (**b**, **d**) of patients with advanced *EGFR*–mutant non-small-cell lung cancer who received mefatinib as first-line therapy at a daily dose of either 60 mg or 80 mg (**a**, **b**) or based on brain metastatic status at presentation (**c**, **d**)

Supplementary Figure S1 summarizes the study design. The phase Ib study enrolled 34 patients who were non-randomized/assigned to receive 60 mg mefatinib (*n* = 15) or 80 mg mefatinib (*n* = 19). The phase II study enrolled a total of 72 patients who were randomized to receive either 60 mg mefatinib (*n* = 36) or 80 mg mefatinib (*n* = 36). In total, 51 patients (48.1%, 51/106) received a lower dose (60 mg) and 55 patients (51.9%, 55/106) received a higher dose (80 mg) of mefatinib. The patients who received 60 mg had an ORR of 80.4%, disease control rate (DCR) of 96.1%, PFS of 15.1 months, and OS of 30.6 months. As compared to 60 mg, the patients who received 80 mg had a numerically higher ORR (89.1%) and DCR (98.2%) but a close PFS (15.4 months) and OS (32.1 months) (Fig. 1a, b). Collectively, the cohort achieved an ORR of 84.9%, DCR of 97.2%, median PFS of 15.4 months, and median OS of 31.6 months. Table 2 summarizes the clinical outcomes of mefatinib.

**Table 2:** Clinical outcomes of the cohort

Clinical outcomes	All patients (*n* = 106)	60 mg (*n* = 51)	80 mg (*n* = 55)
Treatment outcomes; *n* (%)			
Partial response	90 (84.9%)	41 (80.4%)	49 (89.1%)
Stable disease	13 (12.3%)	8 (15.7%)	5 (9.1%)
Progressive disease	2 (1.9%)	2 (3.9%)	0
Unknown	1 (0.9%)	0	1 (1.8%)
Objective response rate; % (95% CI)	84.9% (76.6%, 91.1%)	80.4% (66.9%, 90.2%)	89.1% (77.8%, 95.9%)
Disease control rate; % (95% CI)	97.2% (92.0%, 99.4%)	96.1% (86.5%, 99.5%)	98.2% (90.3%, 100.0%)
Survival outcomes; median (95% CI)			
Median PFS (months)	15.4 (12.9, 17.9)	15.1 (11.7, 18.5)	15.4 (10.5, 20.3)
Median OS (months)	31.6 (26.4, 36.8)	30.6 (24.0, 37.2)	32.1 (27.0, 37.2)

*CI* confidence intervals, *PFS* progression-free survival, *OS* overall survival

We also analyzed the clinical outcomes of the patients according to their brain metastasis status at presentation. With first-line mefatinib, patients with brain metastasis achieved an ORR of 87.1% and DCR of 96.8%, while patients with no brain metastasis at baseline achieved an ORR of 82.4% and DCR of 97.3% (Supplementary Table S1). Although treatment outcomes were similar, patients with brain metastasis (*n* = 31) had significantly shorter PFS (12.8 vs. 18.5 months; HR: 0.442; *p* = 0.002; Fig. 1c) and OS (25.2 vs. 32.2 months; HR: 0.552; *p* = 0.03; Fig. 1d), suggesting that brain metastasis at baseline is a negative prognostic factor.

### Safety profile

All the 106 patients were evaluable for safety profile. Mefatinib at both doses tested was generally well-tolerated at the first-line setting. In patients treated with 60 mg of mefatinib, common grade ≥ 3 treatment-related adverse events observed were rash (13.8%; *n* = 7), diarrhea (11.8%; *n* = 6), decreased appetite (5.9%; *n* = 3), ureterolithiasis (3.9%; *n* = 2), and anemia (3.9%; *n* = 2). Among the patients treated with 80 mg of mefatinib, diarrhea (27.3%; *n* = 15), rash (20.0%; *n* = 11), mouth ulceration (7.3%; *n* = 4), and stomatitis (7.3%; *n* = 4) were the commonly observed grade ≥ 3 treatment-related adverse events. Mefatinib dose reduction was necessary on 31 events for 16 patients (15.1%; 16/106) due to rash (5.7%; *n* = 6; 7 events), diarrhea (3.8%; *n* = 4; 8 events); paronychia (2.8%; *n* = 3; 5 events), and stomatitis (2.8%; *n* = 3; 3 events). Only two patients who received the initial mefatinib dose of 60 mg required dose reduction (3.9%; 2/51; 5 events), while dose reductions were necessary for 14 patients (25.5%; 14/55; 26 events) who received 80 mg mefatinib. Two patients (1.9%; 2/106) who each received 60 mg and 80 mg had grade IV rash that resulted in treatment termination. No fatal adverse events and unexpected drug-related toxicity were observed in both cohorts. Table 3 summarizes the adverse events reported by >10% of the study cohort.

**Table 3:** Adverse events reported in >10% of the patients

Adverse events	All patients (*n* = 106)				60 mg (*n* = 51)				80 mg (*n* = 55)			
	Grade ≥ 3		Any grade		Grade ≥ 3		Any grade		Grade ≥ 3		Any grade	
	No.	%	No.	%	No.	%	No.	%	No.	%	No.	%
Diarrhoea	21	19.8	100	94.3	6	11.8	48	94.1	15	27.3	52	94.5
Rash	18	17.0	92	86.8	7	13.7	42	82.4	11	20.0	50	90.9
Paronychia	4	3.8	57	53.8	2	3.9	20	39.2	2	3.6	37	67.3
Weight decreased	2	1.9	51	48.1	1	2.0	24	47.1	1	1.8	27	49.1
Mouth ulceration	5	4.7	39	36.8	1	2.0	21	41.2	4	7.3	18	32.7
Decreased appetite	4	3.8	37	34.9	3	5.9	19	37.3	1	1.8	18	32.7
Anaemia	4	3.8	36	34.0	2	3.9	13	25.5	2	3.6	23	41.8
Ureterolithiasis	2	1.9	33	31.1	0	0	0	0	2	3.6	19	34.5
Stomatitis	5	4.7	31	29.2	1	2	12	23.5	4	7.3	19	34.5
Upper respiratory tract infection	0	0	31	29.2	0	0	14	27.5	0	0	17	30.9
Dry skin	1	0.9	27	25.5	0	0	14	27.5	1	1.8	13	23.6
Vomiting	1	0.9	26	24.5	0	0	11	21.6	1	1.8	15	27.3
Alopecia	0	0	24	22.6	0	0	13	25.5	0	0	11	20.0
Nausea	0	0	24	22.6	0	0	10	19.6	0	0	14	25.5
Hypokalaemia	4	3.8	23	21.7	2	3.9	13	25.5	2	3.6	10	18.2
Asthenia	0	0	22	20.8	0	0	11	21.6	0	0	11	20.0
Conjunctivitis	1	0.9	20	18.9	0	0	10	19.6	1	1.8	10	18.2
Urinary tract infection	1	0.9	19	17.9	0	0	7	13.7	1	1.8	12	21.8
Palmar-plantar erythrodysaesthesia syndrome	1	0.9	17	16.0	0	0	0	0	0	0	12	21.8
Angular cheilitis	0	0	16	15.1	0	0	6	11.8	0	0	10	18.2
Nasal dryness	0	0	15	14.2	0	0	0	0	0	0	10	18.2
Hepatic function abnormal	0	0	14	13.2	0	0	9	17.6	0	0	0	0
Alanine aminotransferase increased	1	0.9	13	12.3	1	2	7	13.7	0	0	6	10.9
Hypoalbuminaemia	1	0.9	12	11.3	1	2	6	11.8	0	0	6	10.9
Insomnia	0	0	12	11.3	0	0	7	13.7	0	0	0	0
Skin fissures	0	0	12	11.3	0	0	0	0	0	0	7	12.7
Constipation	0	0	11	10.4	0	0	6	11.8	0	0	0	0
Proteinuria	0	0	11	10.4	0	0	6	11.8	0	0	0	0
Supraventricular extrasystoles	0	0	11	10.4	0	0	0	0	0	0	6	10.9

### Potential predictive molecular biomarkers

We first investigated the survival outcomes based on specific baseline *EGFR* sensitizing mutations. Supplementary Figure S2 summarizes the mutation profile of the 69 patients who had baseline plasma samples submitted for NGS-based mutation profiling. Of them, 59.4% (41/69) had *EGFR* 19del and 40.6% (28/69) had L858R. Patients harboring *EGFR* 19del had similar PFS (*p* = 0.899, HR: 0.961, 95% CI: [0.518–1.783]) and OS (*p* = 0.083, HR: 0.555, 95% CI: [0.285–1.08]) as compared to patients harboring *EGFR* L858R (Supplementary Fig. S3a). Among the patients harboring *EGFR* 19del, PFS (*p* = 0.72) and OS (*p* = 0.37) were comparable for those with common E746_A750del (*n* = 30) and uncommon non-E746_A750del (*n* = 11) (Supplementary Fig. S3b).

**Fig. 2:**
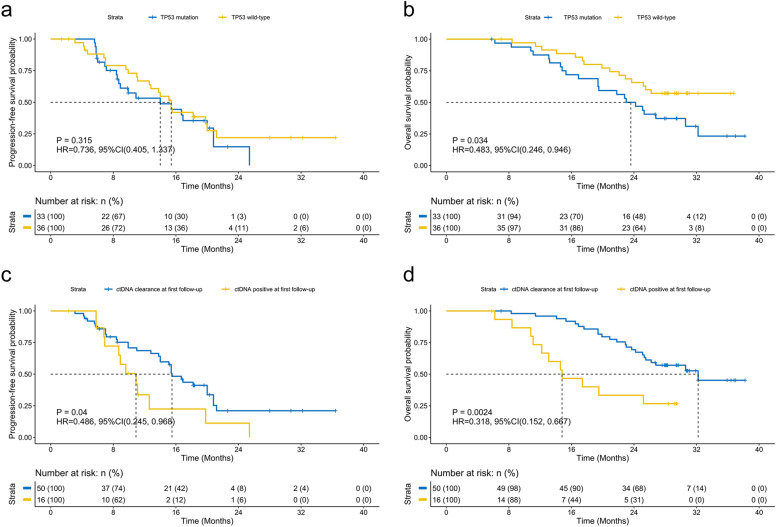
Kaplan–Meier curves comparing the PFS (**a**) and OS (**b**) of patients with advanced *EGFR*–mutant non-small-cell lung cancer who had concomitant *TP53* mutations as compared to patients who had wild-type *TP53*. Patients who experienced ctDNA clearance at first follow-up had significantly longer survival outcomes. Kaplan–Meier curves comparing the PFS (**c**) and OS (**d**) of patients with *EGFR*–mutant non-small-cell lung cancer who experienced ctDNA clearance at first follow-up compared with patients who had detectable mutations

We then investigated the impact of certain concurrent mutations at baseline on survival outcomes. The most common concurrent mutations in the cohort were *TP53* and *EGFR* amplifications, identified from 47.8% (33/69) and 20.3% (14/69) of the patients, respectively (Supplementary Fig. S2). In our cohort, patients harboring any concurrent *TP53* mutations (*n* = 33) (14.0 vs 15.4 months; *p* = 0.315, HR: 0.736, 95% CI: [0.405–1.337]; Fig. 2a) or those harboring *TP53* mutations in exons 5–8 (*n* = 27) (*p* = 0.49, HR: 0.802, 95% CI: [0.428–1.502]; Supplementary Fig. S4a) had a comparable PFS as patients having wild-type *TP53*. The OS for both groups have not been reached (*p* = 0.034, HR: 0.483, 95% CI: [0.246–0.946]; Fig. 2b; *p* = 0.067, HR: 0.514, 95% CI: [0.252–1.048]; Supplementary Fig. S4a). Patients with concurrent mutations in genes involved in DNA damage repair (DDR) at baseline had similar PFS for first-line mefatinib therapy (*p* = 0.18, HR: 0.515, 95% CI: [0.197–1.347]) but significantly shorter OS (*p* = 0.004, HR: 0.265, 95% CI: [0.108–0.649]) as compared to those who were wild-type for these genes (Supplementary Fig. S4b). Supplementary Table S2 summarizes the list of genes related to the DDR pathway included in the analysis. Moreover, patients with or without concurrent copy number variations (CNV), including *EGFR* amplifications, had statistically similar PFS (CNV, *p* = 0.092, HR: 0.571, 95% CI: [0.297–1.096]; Supplementary Fig. S4c; EGFRamp, p = 0.14, HR: 0.595, 95% CI: [0.298–1.187]; Supplementary Fig. S4d) but significantly shorter OS (CNV, *p* = 0.008, HR: 0.389, 95% CI: [0.193–0.786]; Supplementary Fig. S4c; *EGFR* amplifications, *p* = 0.018, HR: 0.412, 95% CI: [0.197–0.856]; Supplementary Fig. S4d). These data suggest that despite harboring concurrent mutations that are correlated with shorter EGFR-TKI response and/or poor overall prognosis, the patients who harbor these concurrent mutations derive similar survival benefit from first-line mefatinib therapy as their wild-type counterpart.

In addition to baseline molecular factors, we further investigated the dynamic changes in mutations from baseline until the first follow-up 6 weeks after initiating mefatinib therapy (F1) to understand whether their detection or the lack thereof could predict efficacy and survival outcomes. The mutation detection rates and the maximum allelic fraction (MaxAF) at F1 were dramatically reduced compared with baseline levels (Supplementary Fig. S5). MaxAF is defined as the highest allelic fraction among all the mutations detected from the patient sample. At F1, patients with ctDNA clearance, defined as having no mutation detected from the panel used, were associated with significantly longer PFS (*p* = 0.04, HR: 0.486, 95% CI: [0.245–0.968]; Fig. 2c) and OS (*p* = 0.0024, HR: 0.318, 95% CI: [0.152–0.667]; Fig. 2d) as compared to patients who had mutations detected. Consistently, patients who experienced clearance of *EGFR* sensitizing mutations at F1 had a trend of longer PFS (*p* = 0.094, HR: 0.465, 95% CI: [0.189–1.14]) and significantly longer OS (*p* < 0.001, HR: 0.219, 95% CI: [0.092–0.522] than patients who retained their *EGFR* sensitizing mutations (Supplementary Fig. S6a). Notably, 50 (75.8%) and 55 (87.3%) patients had ctDNA clearance and clearance of *EGFR* mutation at F1, respectively. Among the patients who achieved PR, the number of patients who had ctDNA clearance was numerically higher than those who remained ctDNA positive; however, it did not reach statistical significance (72.7% (40/55) vs 27.3% (15/55); *p* = 0.27). The patients who achieved PR as best response and experienced ctDNA clearance at first follow-up had statistically similar PFS (*p* = 0.064, HR: 0.513, 95% CI: [0.253–1.039]) but had significantly longer OS (*p* = 0.007, HR: 0.334, 95% CI: [0.151–0.739]) than patients who were ctDNA positive (Supplementary Fig. S6b).

### Molecular mechanisms of acquired resistance to mefatinib

**Fig. 3:**
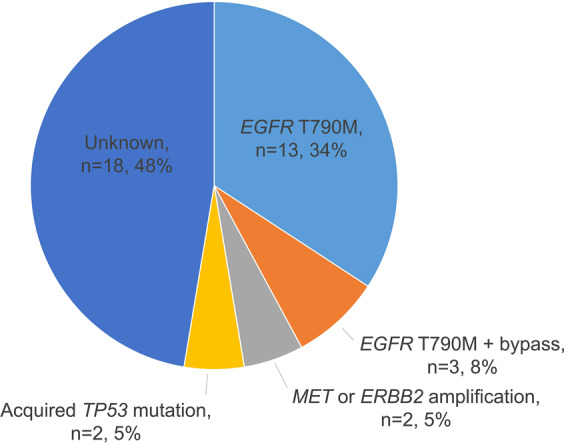
Distribution of molecular mechanisms of acquired resistance to mefatinib. Pie chart illustrating the distribution of acquired mutations identified in the cohort at the progression from mefatinib therapy

Finally, the mutation profiles at baseline and disease progression were compared to elucidate the molecular mechanisms of acquired mefatinib resistance. EGFR-TKI progression was associated with the emergence of new mutations or the increase in the allelic fraction of *EGFR* sensitizing mutation or MaxAF.^29^ Consistently, in our cohort, the mutation detection rate and MaxAF had an increasing pattern when PD was confirmed (Supplementary Fig. S5). A total of 38 patients experienced PD, with most of them detected with *EGFR* T790M (42.1%, 16/38). Three patients who acquired *EGFR* T790M also had concurrent bypass resistance mechanisms such as *BRAF* V600E (*n* = 2) and *MET* amplification (*n* = 1). Other acquired resistance mechanisms detected from the cohort included *ERBB2* amplification (*n* = 1), *MET* amplification (*n* = 1), and *TP53* mutations (*n* = 2). The remaining 18 patients had no known resistance mechanisms detected. Figure 3 illustrates the distribution of molecular mechanisms of acquired resistance for mefatinib detected from our cohort.

## Discussion

Mefatinib, with chemical formula: C_29_H_27_ClF_3_N_5_O_10_ and molecular weight of 698 g/mol, is a second-generation, irreversible, highly effective dual inhibitor of EGFR/HER2 (Supplementary Fig. S7). The half-maximal inhibitory concentration (IC50) of mefatinib for EGFR kinase is 0.4 nanomolar (nM) and for HER2 kinase is 11.7 nM. Using nude mouse xenograft models of erlotinib-resistant human NSCLC NCI-H1975 harboring *EGFR* L858R/T790M double mutation, mefatinib demonstrated similar or even better anti-tumor activity as compared with afatinib at the same dose. In the nude mouse xenograft model of human lung adenocarcinoma HCC827, the lowest effective dose of 0.2 mg/kg for mefatinib achieved 99.6% suppression of tumor growth, while the comparator group treated with erlotinib at a dose of 12.5 mg/kg only achieved 73.1% tumor growth inhibition. Mefatinib has good drug metabolism, pharmacokinetics (PK), and oral bioavailability. After oral administration, mefatinib is quickly distributed to various tissues of the body, and highly distributed in the lung, small intestine, and stomach. The PK/pharmacodynamic test in rats showed that mefatinib is quickly distributed into tumor tissues after oral administration. Mefatinib metabolism undergoes N-demethylation, sulfation, and glutathione conjugation, with the metabolites concentrated in feces and undetected in plasma, bile, and urine. Mefatinib has no obvious inducing effect on rat liver microsomal cytochrome P450 enzymes. Safety pharmacology shows that mefatinib has no significant effect on the central nervous system function of mice, the cardiovascular, respiratory system, and body temperature of awake cynomolgus monkeys. At the same time, the IC50 value of hERG potassium channel inhibition is 27.86 mM, which is ~100 times higher than the expected maximum serum concentration (Cmax) (daily dose of 100 mg). Toxicity tests of repeated administration in rats, dogs, and cynomolgus monkeys showed that the gastrointestinal tract and kidney are the main target organs for toxicity, consistent with other approved EGFR inhibitors. In addition, in vivo and in vitro studies with mefatinib have shown no teratogenicity and reproductive toxicity.

Our present study reports the results of the phase Ib/II clinical trial of mefatinib as first-line treatment of patients with *EGFR-*mutant locally advanced/advanced NSCLC. Our study achieved its primary and secondary objectives. Mefatinib at either dose of 60 or 80 mg once daily achieved an ORR of 84.9%, DCR of 97.2%, median PFS of 15.4 months, and median OS of 31.6 months in the first-line treatment of patients with *EGFR*-mutant stage IIIB-IV NSCLC. Our study has demonstrated a better efficacy for mefatinib than the reported clinical outcomes of afatinib in two phase III studies, LUX-Lung 3 (*n* = 345; ORR 56%, median PFS 11.1 months, median OS 28.2 months) and LUX-Lung 6 (*n* = 364; ORR 67%, median PFS 11.0 months, median OS 23.1 months).^11,12^ Moreover, mefatinib also showed better ORR and PFS than the reported outcomes for first-line dacomitinib from the ARCHER 1050 study (*n* = 227; ORR 75%, median PFS 14.7 months, median OS 34.1 months).^18,23^ Similar to the drug-related toxicity profile of afatinib,^11,12^ the adverse events observed for mefatinib even at a higher dose (80 mg) primarily involved skin (i.e., rash and stomatitis) and gastrointestinal (i.e., diarrhea) toxicities, which were well-tolerated and manageable. As expected, the higher dose of mefatinib had more events of drug-related toxicity than the lower dose. Hence, based on its better safety profile, 60 mg is the recommended daily dose for mefatinib.

Brain metastasis was frequently observed among patients with *EGFR*-mutant than *EGFR*-wild-type NSCLC (29.4% vs 19.7%).^30^ Subgroup analyses of patients with brain metastasis from the LUX-Lung 3 and LUX-Lung 6 have demonstrated a PFS of 11.1 months and 8.2 months, respectively, for first-line afatinib therapy.^31^ As compared to afatinib, our cohort demonstrated better clinical outcomes with mefatinib therapy with a PFS of 12.8 months, suggesting that first-line mefatinib is a good treatment option for patients who presented with brain metastasis.

Anti-tumor activity of first- and second-generation EGFR-TKI could vary depending on the specific *EGFR* sensitizing mutation, wherein NSCLCs harboring *EGFR* L858R demonstrate poorer clinical outcomes to gefitinib, erlotinib, and afatinib than those harboring 19del.^4,6,22,32^ Meanwhile, patients with common *EGFR* 19del E746_A750del and uncommon non-E746_A750del also demonstrate distinct EGFR-TKI sensitivity and survival outcomes.^33,34^ Interestingly, with mefatinib therapy, common *EGFR* sensitizing mutations did not affect the efficacy as shown by the statistically similar survival outcomes of patients with *EGFR* 19del and L858R and patients with different *EGFR* 19del subtypes (Supplementary Fig. S3). These findings suggest that mefatinib effectively and non-selectively inhibits various subtypes of *EGFR*-mutant lung cancer resulting in similar clinical benefits for patients regardless of *EGFR* sensitizing mutation types. The main advantage of using NGS in mutational profiling at baseline is its ability to reflect intratumor genetic heterogeneity by simultaneously detecting concurrent somatic mutations associated with treatment response prediction. *TP53* mutations are the most common concurrent mutations in NSCLC, accounting for 30–72%, and are associated with poor clinical outcomes with EGFR-TKI therapy.^24–28^ In our cohort, we found statistically similar PFS outcomes between patients with wild-type *TP53* and those who harbor *TP53* mutations, even those located in exons 5–8 (Supplementary Fig. S5a), indicating that mefatinib might be a more suitable therapeutic option for patients with concurrent *TP53* mutation. In addition to *TP53* mutations, other concurrent mutations associated with poor prognosis, including mutations in the DDR pathway, copy number amplifications in any genes, and *EGFR* amplification, also did not affect mefatinib efficacy. This finding suggests that patients with concurrent gene mutations benefit from mefatinib therapy and therapeutic decisions on their subsequent-line therapy after mefatinib progression are crucial in improving their prognosis. In addition to baseline mutation profiling, serial analysis of plasma samples during treatment could also provide information on the molecular changes associated with therapeutic response or resistance. Numerous studies have demonstrated the association between better clinical response and survival outcomes of patients who experienced ctDNA clearance and either decrease or clearance of *EGFR* sensitizing mutations during EGFR-TKI therapy.^29,35–39^ Consistently, the patients in our cohort who had ctDNA and *EGFR* sensitizing mutation clearance within 6 weeks after initiating mefatinib therapy had a better overall prognosis than patients who remained ctDNA mutation-positive.

At progression, the emergence of additional mutations is implicated in resistance to therapy and tumor progression.^29,35,40^ The molecular mechanisms of acquired resistance with mefatinib were predominantly *EGFR* T790M, consistent with other commercially available first- and second-generation EGFR-TKI.^6,14,15,40,41^ Other common bypass mechanisms, including *MET* and *ERBB2* amplifications, were also identified in our cohort. In general, our analysis demonstrated that mefatinib had a similar profile of acquired resistance as compared to first- and second-generation EGFR-TKIs, particularly the acquisition rate of *EGFR* T790M. This finding indicates that after developing resistance from first-line mefatinib, osimertinib, and other therapeutic strategies or guidelines currently in use for managing NSCLC after progression from prior generations of EGFR-TKI remains applicable after mefatinib progression. However, this investigation on resistance mechanisms was only limited to the molecular mechanisms of acquired resistance detected from plasma samples and did not include the analysis of tissue rebiopsy samples. An analysis of the rate of histological transformations after mefatinib progression, particularly among the subset of patients who had unknown molecular resistance mechanisms, could be clinically relevant and should be explored in future studies.

Recent reports have demonstrated the superior clinical activity of afatinib in targeting uncommon *EGFR* mutations, including single G719X, L861Q, and S7681, and compound mutations.^42,43^ A phase II trial is currently ongoing to explore the activity of mefatinib in targeting uncommon *EGFR* mutations (ChiCTR2000029058). Our present study is limited to investigating the clinical outcomes of patients with common *EGFR* sensitizing mutations such as 19del and L858R. A prospective, double-blind, randomized, controlled phase III study with a larger cohort that compares the efficacy of mefatinib with gefitinib is currently ongoing (ChiCTR2000028763). We anticipate the results from these two clinical studies to further establish the efficacy of mefatinib as first-line therapy of patients with advanced *EGFR*-mutant NSCLC.

In conclusion, our study provides clinical evidence that mefatinib is an effective and well-tolerable therapeutic option for the first-line treatment of patients with advanced *EGFR*-mutant NSCLC. Mefatinib might benefit patients with concurrent *TP53* mutations as well as those with brain metastasis at presentation. CtDNA clearance within six weeks of mefatinib treatment was associated with better survival outcomes. Furthermore, mefatinib had similar molecular mechanisms of acquired resistance than other EGFR-TKI, indicating the applicability of currently available therapeutic strategies and guidelines for managing advanced NSCLCs after EGFR-TKI progression.

## Patients and methods

### Clinical trial registration

The Phase Ib (ChiCTR2000029062) and phase II (ChiCTR2000029059) studies on mefatinib were registered on the Chinese Clinical Trial Registry.

### Patient inclusion and exclusion criteria

Patients newly diagnosed with *EGFR*-mutant stage IIIB-IV NSCLC from five participating centers from May 2017 to June 2018 for the phase Ib study and July 2018 to December 2018 for the phase II study were enrolled in this study. The phases Ib and II aimed to investigate and compare two mefatinib doses (60 and 80 mg), which were selected based on the results of the phase Ia dose-escalation study. The main objective of the phase Ib study was to investigate and compare the ORR and safety between the two mefatinib doses and included 34 patients who were non-randomly assigned to each dose group, with 15 patients assigned to receive 60 mg and 19 patients assigned to receive 80 mg. Meanwhile, the main objective of the phase II part of this study was to investigate the efficacy and safety of long-term administration of mefatinib and compare the PFS between the two mefatinib dose groups. A total of 72 patients were enrolled and randomized to receive either of the two mefatinib doses. *EGFR* status was assessed using amplification refractory mutation system polymerase chain reaction (ARMS-PCR). Inclusion criteria included: (1) Having cytologically or histologically confirmed NSCLC; (2) Unresectable, locally advanced (stage IIIB/IIIC) or metastatic disease (stage IV); (3) Having confirmed *EGFR* mutation status (i.e., 19del or L858R) of tissue biopsy samples using ARMS-PCR; (4) Having at least 1 unidimensional measurable lesion as per Response Evaluation Criteria in Solid Tumors (RECIST) version 1.1; (5) Having an Eastern Cooperative Oncology Group (ECOG) performance status of 0–1; (6) Have not received previous systemic therapy; (7) Signed informed consent. Exclusion criteria included: (1) Concurrent mutation in oncogenic drivers including *ALK*, *BRAF*, *ERBB2*, *KRAS*, *MET*, *RET*, and *ROS1*; (2) Prior local and systemic anti-tumor therapy including chemotherapy, EGFR-TKIs, angiogenesis inhibitors, and immunotherapy; (3) Other clinical factors that were deemed unsuitable for this study. All patients were required to provide written informed consent before enrollment. The study was performed in accordance with the Declaration of Helsinki and its amendments. The study protocol was approved by the Ethics committee of all the participating hospitals.

### Treatment schedule and evaluation of treatment response and adverse events

Patients were instructed to take mefatinib at either 60 mg or 80 mg orally once daily. Comprehensive examination, including history taking, physical evaluation, blood and urine laboratory testing, and radiologic imaging (computed tomography or magnetic resonance imaging), was performed at screening, at follow-up visits every six weeks, and at treatment termination. Treatment response was assessed by the investigators according to the RECIST version 1.1 criteria.^44^ Treatment-related adverse events were evaluated according to the National Cancer Institute Common Terminology Criteria for Adverse Events (CTCAE) version 4.0.3. Reported adverse events were graded based on severity, with grade 1 as the mildest and grade 5 as death. Dose reductions were permitted for intolerable drug-related toxicities.

### Study endpoints

For the phase Ib part of the study, ORR was the primary efficacy endpoint and treatment-related adverse events were the secondary endpoint. Based on the promising efficacy and manageable toxicity of both mefatinib doses in the phase Ib study, we pursued to investigate the efficacy and safety of long-term administration of both doses of mefatinib in a multi-center, randomized, open-label phase II study. The primary objective of the phase II study was to assess PFS. The secondary endpoints were ORR, DCR, OS, and adverse events. ORR was defined as the proportion of patients who achieved complete response (CR) and partial response (PR). DCR was defined as the proportion of the patients who achieved CR, PR, and stable disease (SD). PFS was defined as the time from the start of mefatinib treatment until the treatment termination or the day of the last follow-up. OS was defined as the time from the start of mefatinib treatment until the date of death or the last day of follow-up. Surviving patients at the time of data cutoff were censored for OS as of the last follow-up date. The data cutoff date was 24 March 2021.

### Next-generation sequencing (NGS)-based mutation profiling

For the biomarker analyses, longitudinal blood samples were obtained from the patients to elucidate the somatic mutation profile at each time point using NGS. Approximately 10 ml of blood samples were obtained from the patients at each of the three-time points: at baseline before receiving mefatinib therapy, at first follow-up 6 weeks after starting mefatinib therapy (cycle 1), and at the time when disease progression is radiologically confirmed (PD). Plasma samples were submitted for capture-based targeted genomic sequencing to Burning Rock Biotech, a commercial clinical laboratory accredited by the College of American Pathologists and certified by the Clinical Laboratory Improvement Amendments. Targeted genomic sequencing was performed using a panel consisting of 168 lung cancer-related genes (Lung Plasma, Burning Rock Biotech, Guangzhou, China) following optimized protocols as described previously.^45,46^ The data for the exploratory biomarker study are included as Supplementary data S1.

### Statistical methods

Statistical analyses were performed using R software (R version 3.5.3; Vienna, Austria; RRID:SCR_001905). Descriptive statistical analysis was performed to evaluate the safety and efficacy without statistical testing. The quantitative parameters were described as the number and percentage of cases with the categorical variables described as median with range. All analyses were summarized by dose. The DCR and ORR were presented as means and 95% confidence intervals (CI) for each dose group. The Kaplan–Meier method was used to estimate the median PFS and OS and their corresponding 95% CI. Cox proportional hazards regression analyses were applied to the survival analyses to calculate the corresponding hazard ratios (HR) and 95% CI and compare the survival outcomes between groups using baseline brain metastatic status as the adjustment covariate. P values <0.05 were defined as statistically significant.

## Supplementary information

Supplementary Materials

Dataset 1

## Data Availability

All authors confirm adherence to the policy. The data that support the findings of the exploratory biomarker study are included as Supplementary data S1. Supplementary data S1 includes individual participant data that underlie the results of the biomarker study reported in the article after de-identification. The data will be available upon publication with no end date to anyone who wishes to access the data. Correspondence and requests for additional data should be addressed to KW.
