# Supplementary material for: Mefatinib as first-line treatment of patients with advanced EGFR-mutant non-small-cell lung cancer: a phase Ib/II efficacy and biomarker study
Source: Signal Transduct Target Ther. 2021 Nov 1;6:374. doi: 10.1038/s41392-021-00773-3 (PMC8558340; doi:10.1038/s41392-021-00773-3)
Supplement: Supplementary file 1 — Supplementary Materials [file 41392_2021_773_MOESM1_ESM.docx]

Supplementary Materials for

**Mefatinib as First-line Treatment of Patients with Advanced *EGFR*-mutant Non-small-cell Lung Cancer: A Phase Ib/II Efficacy and Biomarker Study**

Pingli Wang, Yuping Li, Dongqing Lv, Lingge Yang, Liren Ding, Jianya Zhou, Wei Hong, Youfei Chen, Dongqing Zhang, Susu He, Jianying Zhou*, Kai Wang*

Correspondence to: Kai Wang (Email: Kaiw@zju.edu.cn); Jianying Zhou (Email: zjyhz@zju.edu.cn)

**This PDF file includes**

Figures S1 to S7

Table S1 to S2

**
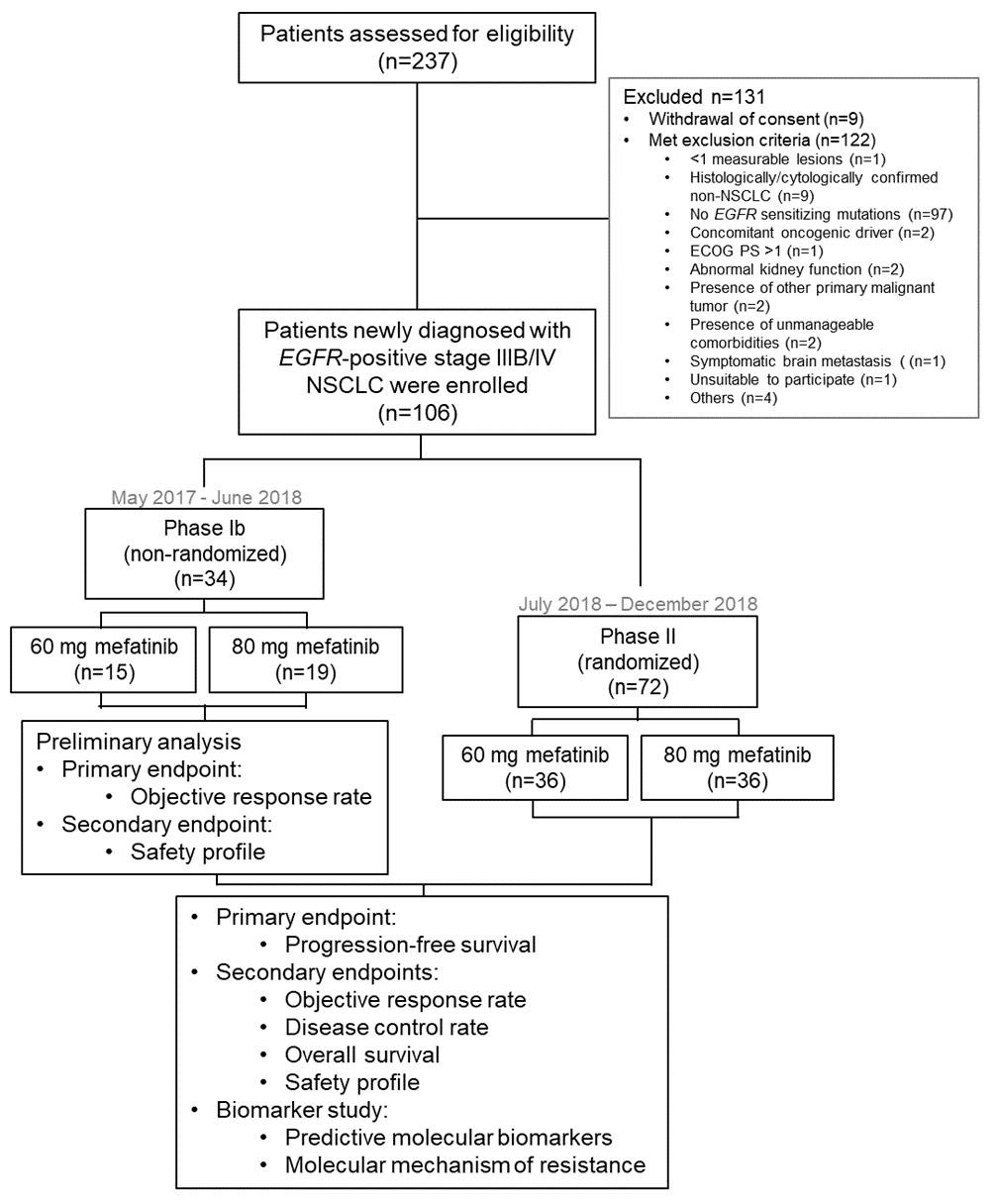
**

**Figure S1**. CONSORT diagram illustrating the study design

**Figure S2**. Baseline somatic mutation profile of the 69 patients who submitted plasma samples for NGS.


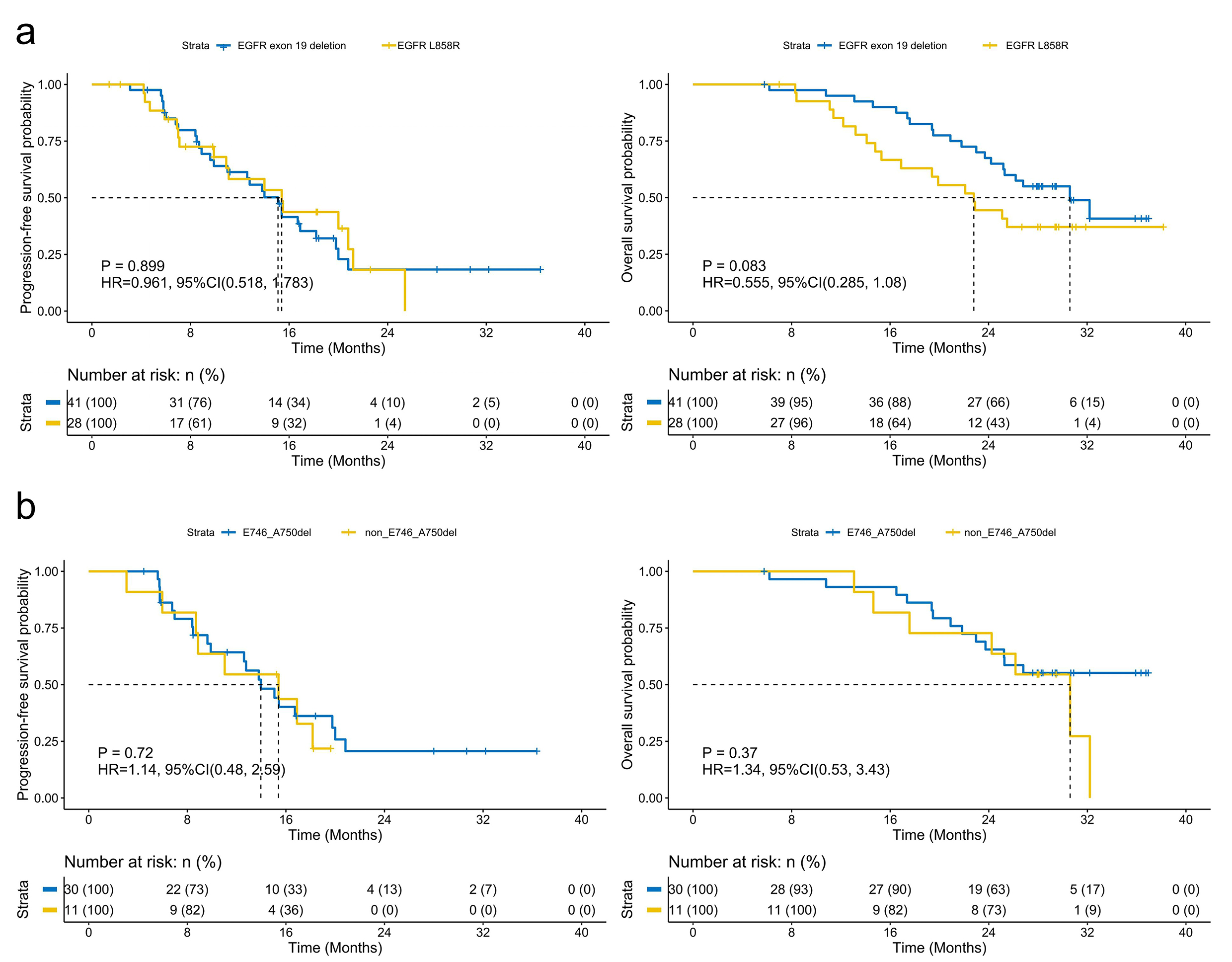


**Figure S3**. Survival outcomes with mefatinib were similar for patients harboring *EGFR* exon 19 deletion and *EGFR* L858R (**a**) and for patients having *EGFR* exon 19 deletion variants E746_A750del and non E746_A750del (**b**).


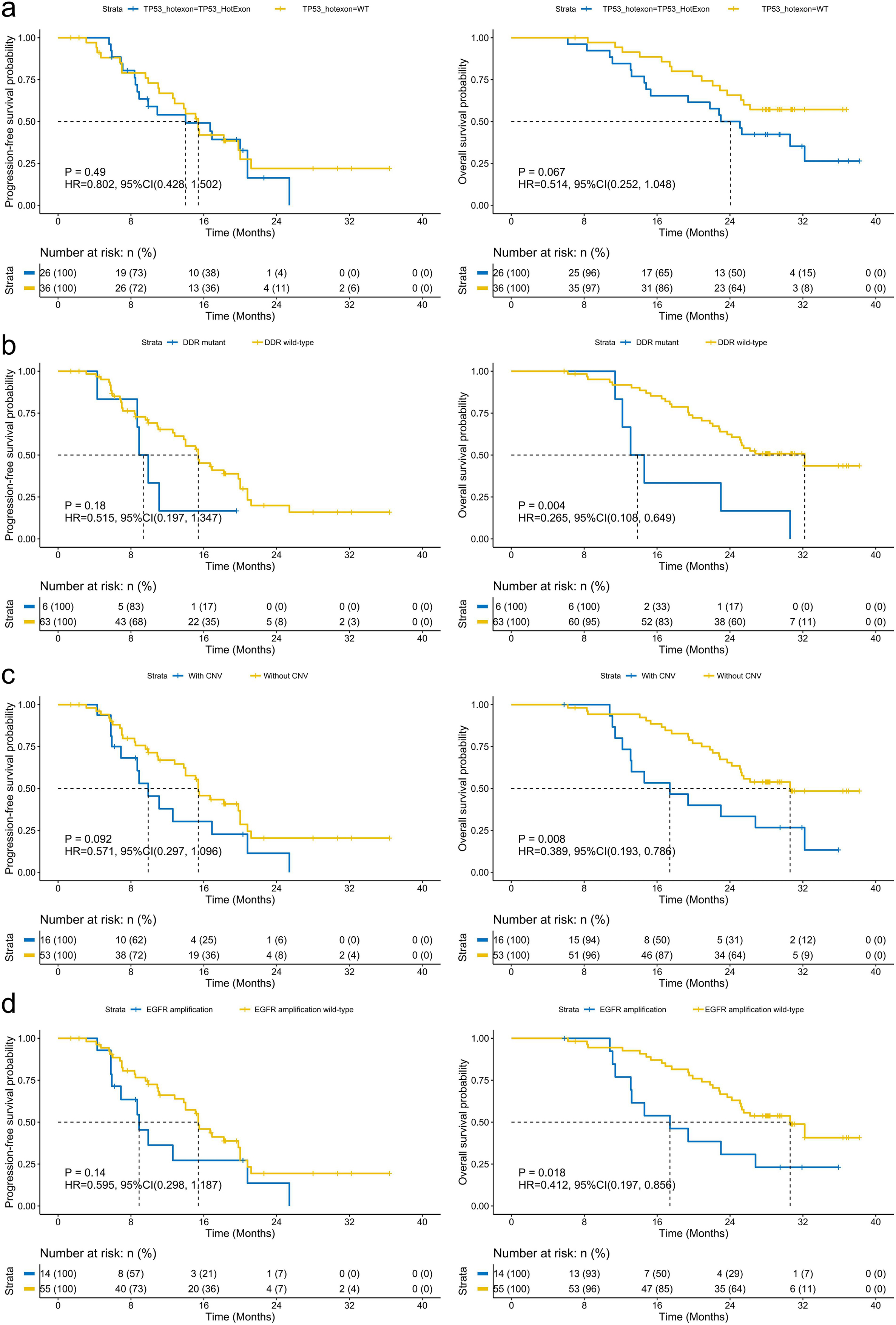


**Figure S4.** Patients harboring concurrent mutations in (**a**) *TP53* located in exons 5-8 (hot exons), (**b**) genes involved in the DNA damage repair (DDR) pathway, (**c**) copy number variations (CNV), or (**d**) *EGFR* amplification who received first-line mefatinib had comparable progression-free survival as those who were wild-type for these genes.


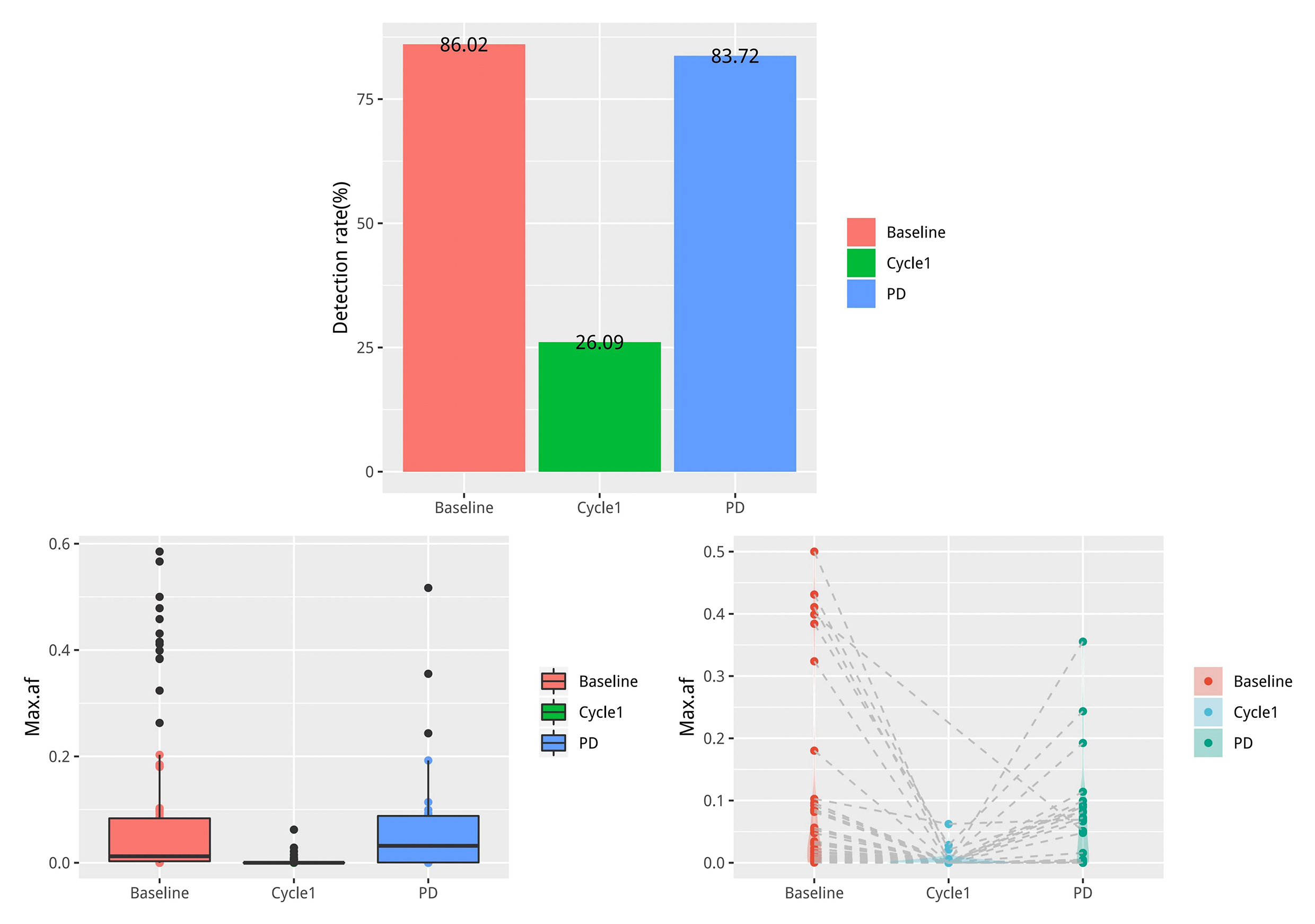


**Figure S5**. Dynamics of mutation detection rate and maximum allelic fraction (MaxAF) at baseline, at first follow-up 6 weeks after initiating mefatinib therapy (cycle 1), and confirmation of disease progression (PD).


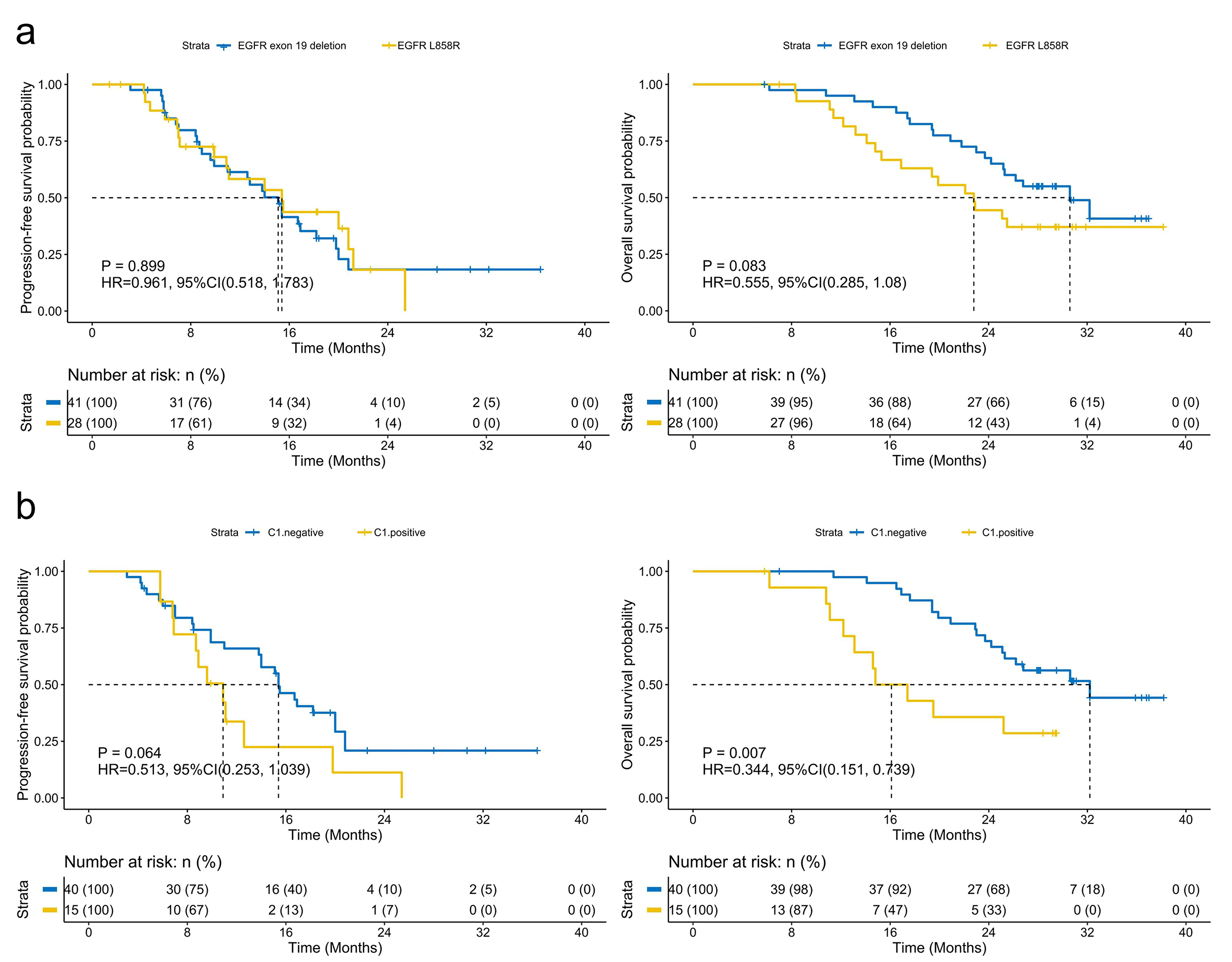


**Figure S6**. Molecular dynamics at first-follow up was associated with better prognosis. Patients who had (**a**) undetected *EGFR* sensitizing mutations at first follow-up or (**b**) those who achieved partial response to first-line mefatinib and experienced ctDNA clearance at first follow-up had significantly longer survival outcomes.


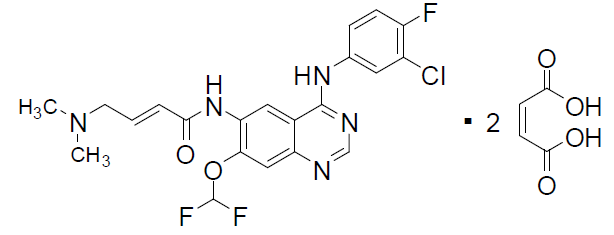


**Figure S7**. Chemical structure of mefatinib (chemical formula: C29H27ClF3N5O10; molecular weight: 698 g/mol).

**Table S1.** Clinical outcomes of the cohort based on brain metastasis status at presentation

| Clinical outcomes | All patients (n=106) | With brain metastasis at baseline (n=31) | Without brain metastasis at baseline (n=74) |
| --- | --- | --- | --- |
| Treatment outcomes; n (%) | | | |
| Partial response | 90 (84.9%) | 27 (87.1%) | 61 (82.4%) |
| Stable disease | 13 (12.3%) | 3 (9.7%) | 11 (14.9%) |
| Progressive disease | 2 (1.9%) | 1 (3.2%) | 1 (1.4%) |
| Unknown | 1 (0.9%) | 0 | 1 (1.4%) |
| Objective response rate; % (95% CI) | 84.9% (76.6%, 91.1%) | 87.1% (70.2%, 96.4%) | 82.4% (71.8%, 90.3%) |
| Disease control rate; % (95% CI) | 97.2% (92.0%, 99.4%) | 96.8% (83.3%, 99.9%) | 97.3% (90.6%, 99.7%) |
| Survival outcomes; median (95% CI) | | | |
| Median PFS (months) | 15.4 (12.9, 17.9) | 12.8 (9.9, 15.7) | 18.5 (14.4, 22.6) |
| Median OS (months) | 31.6 (26.4, 36.8) | 25.2(21.7, 28.7) | 32.2 (28.1, 36.3) |

Abbreviations: CI, confidence intervals; PFS, progression-free survival; OS, overall survival; NR, not reached

**Table S2**. List of genes involved in the DNA damage repair (DDR) pathway included in the 168-gene Lung Plasma panel

| Genes | Genes |
| --- | --- |
| *MSH6* | *RAD51D* |
| *MSH2* | *RAD54L* |
| *PMS2* | *MUTYH* |
| *MLH1* | *POLD1* |
| *BRCA1* | *POLE* |
| *BRCA2* | *PARP3* |
| *ATM* | *PARP4* |
| *BRIP1* | *PARP1* |
| *PALB2* | *PARP2* |
| *RAD51C* | *NTHL1* |
| *BARD1* | *RPA1* |
| *CDK12* | *ERCC5* |
| *CHEK1* | *CUL4A* |
| *CHEK2* | *ERCC3* |
| *FANCL* | *ERCC4* |
| *FANCI* | *ERCC1* |
| *PPP2R2A* | *ERCC2* |
| *RAD51B* |  |
